# Comparative Evaluation of Locally Delivered *Aloe vera* Gel as an Adjunct to Scaling and Root Planing in Patients With Periodontitis: A Randomized Clinical Trial

**DOI:** 10.1155/ijod/4088662

**Published:** 2026-07-14

**Authors:** Vinita Boloor, Belim Wasim Siraj, Rajesh Kashyap, Haziel Jenifer, Shruthy Prathap, Abdul Naeem

**Affiliations:** ^1^ Department of Periodontology, Yenepoya Dental College, Yenepoya (Deemed to be University), Mangaluru, Karnataka, India, yenepoya.edu.in

## Abstract

**Background:**

Herbal adjuncts have gained increasing interest in nonsurgical periodontal therapy because of their anti‐inflammatory, antimicrobial, and wound‐healing properties. *Aloe vera* has been explored as a locally delivered therapeutic agent owing to its biological activity and favorable safety profile.

**Aim:**

To evaluate the clinical efficacy of locally delivered *Aloe vera* gel as an adjunct to scaling and root planing (SRP) in patients with Stage II–III, Grade B periodontitis.

**Materials and Methods:**

This randomized split‐mouth clinical trial included systemically healthy patients diagnosed with Stage II–III, Grade B periodontitis. Selected periodontal sites were randomly assigned to receive either SRP with adjunctive subgingival application of locally prepared *Aloe vera* gel or SRP alone. Clinical parameters including plaque index (PI), gingival index (GI), probing pocket depth (PPD), and clinical attachment level (CAL) were recorded at baseline and at 15, 30, and 45 days. An artificial intelligence‐assisted evidence mapping analysis was additionally performed as a supplementary exploratory approach to contextualize the clinical findings within the existing periodontal literature.

**Results:**

Both treatment groups demonstrated statistically significant improvement in all clinical parameters over the study period. At 45 days, sites treated with adjunctive *Aloe vera* gel showed greater reduction in PPD and greater gain in CAL compared with sites treated with SRP alone.

**Conclusion:**

Adjunctive subgingival application of locally prepared *Aloe vera* gel may improve short‐term clinical periodontal outcomes when used alongside conventional nonsurgical periodontal therapy. These findings support its potential role as a safe and economical adjunctive therapeutic option.

## 1. Introduction

Periodontitis is a chronic immune‐inflammatory disease characterized by progressive destruction of the tooth‐supporting structures resulting from a complex interaction between dysbiotic microbial biofilms and the host immune response [1, 2]. Scaling and root planing (SRP) remains the cornerstone of nonsurgical periodontal therapy as it reduces microbial load and inflammation through mechanical disruption of subgingival biofilms. However, complete eradication of periodontal pathogens remains challenging because of anatomical complexities, limited access to deep periodontal pockets, and the potential for microbial recolonization following treatment. According to the 2017 World Workshop Classification, periodontitis is categorized based on staging, which reflects disease severity and complexity of management, and grading, which reflects the rate of disease progression and associated risk factors, thereby providing a more precise framework for diagnosis and treatment planning.

Several local drug delivery systems have been introduced to enhance the therapeutic outcomes of SRP by enabling sustained release of antimicrobial or anti‐inflammatory agents directly into periodontal pockets while minimizing systemic exposure [3]. Although synthetic adjunctive agents such as chlorhexidine and tetracycline have demonstrated clinical benefits, concerns regarding adverse effects, bacterial resistance, and patient compliance have encouraged the exploration of alternative therapeutic options [4].

Herbal substances have received increasing attention in periodontal therapy because of their broad biological properties, biocompatibility, and potential adjunctive therapeutic benefits in inflammatory periodontal diseases [5, 6]. *Aloe vera* (*Aloe barbadensis Miller*) is a medicinal plant recognized for its anti‐inflammatory, antimicrobial, antioxidant, and wound‐healing properties, primarily attributed to biologically active constituents such as anthraquinones, saponins, polysaccharides, and acemannan [7–9]. Previous studies have demonstrated the potential efficacy of *Aloe vera* in reducing gingival inflammation and improving periodontal healing when administered topically [10, 11]. More recent clinical and systematic evidence has further suggested that adjunctive subgingival *Aloe vera* may improve periodontal clinical outcomes when used alongside nonsurgical periodontal therapy [12, 13].

Several clinical trials and systematic reviews have evaluated the adjunctive use of *Aloe vera* in nonsurgical periodontal therapy; however, variations in formulation type, application protocols, delivery methods, and follow‐up duration have resulted in heterogeneous clinical outcomes. In contrast to earlier studies that predominantly employed commercially available preparations or single‐application regimens, the present split‐mouth randomized clinical trial evaluated the effects of repeated subgingival placement of freshly prepared *Aloe vera* gel with controlled retention using periodontal dressing. This protocol was designed to improve local bioavailability and therapeutic persistence within periodontal pockets and to provide additional evidence regarding protocol‐dependent variability in clinical response to herbal adjunctive therapy. In addition, an artificial intelligence‐assisted evidence mapping approach was incorporated as a supplementary exploratory component to contextualize the clinical findings within the evolving body of periodontal literature [14].

## 2. Materials and Methods

The randomized split‐mouth clinical trial was conducted in the Department of Periodontology, Yenepoya Dental College, Yenepoya (Deemed to be University), Mangalore, India, after obtaining approval from the Institutional Ethics Committee (Approval No. YEC2/221). The study protocol adhered to the principles of the Declaration of Helsinki, and written informed consent was obtained from all participants prior to enrollment. The study protocol was initiated prior to the implementation of mandatory prospective clinical trial registration at the institutional level; therefore, the trial was not registered in a public clinical trial registry. However, the study received Institutional Ethics Committee approval before commencement and was conducted in accordance with the Declaration of Helsinki to ensure compliance with accepted ethical standards for human clinical research.

Systemically healthy patients between the ages of 25 and 64 years who were diagnosed with periodontitis were recruited for the study. Periodontal diagnosis and case definition were established according to the 2017 World Workshop Classification of Periodontal and Peri‐Implant Diseases and Conditions. Subjects included in the study presented with stage II–III, Grade B periodontitis, characterized by probing pocket depth (PPD) ≥5 mm and clinical attachment loss ≥3 mm at non‐adjacent sites. The selected age group represented the typical adult population affected by moderate periodontal destruction while minimizing the influence of aggressive disease patterns seen in younger individuals and systemic comorbidities more prevalent in older age groups.

Patients were required to have at least two non‐adjacent affected periodontal sites that met the inclusion criteria. Patients with systemic disease, pregnant or nursing women, smokers, individuals with known hypersensitivity to herbal preparations, those receiving antibacterial or anti‐inflammatory medications, and patients who had undergone periodontal therapy within the previous 6 months were excluded from the study.

A split‐mouth study design was adopted, and selected sites within each subject were randomly assigned to either the test group (SRP with subgingival application of locally prepared *Aloe vera* gel) or the control group (SRP alone) using a computer‐generated random number table. The allocation sequence was generated prior to the initiation of the intervention to ensure unbiased assignment of study sites. Due to the nature of the split‐mouth intervention, operator blinding during treatment was not feasible; however, the examiner responsible for recording clinical parameters remained blinded to the site allocation throughout the study period to minimize detection bias. Participants were not informed about the site allocation to the test or control intervention, thereby maintaining participant‐level blinding. Statistical analysis was performed using coded datasets to minimize the interpretation bias. The study was reported in accordance with the CONSORT guidelines for randomized clinical trials, and a CONSORT flow diagram illustrating participant enrollment, allocation, follow‐up, and analysis is provided.

SRP were performed by a single operator using both ultrasonic and manual instruments under standardized clinical conditions. In the test sites, ~0.2 mL of freshly prepared *Aloe vera* gel was delivered subgingivally 15 min after completion of SRP using a sterile syringe, followed by placement of periodontal dressing to enhance local retention for 24 h. The volume of 0.2 mL per site was selected to ensure adequate filling of periodontal pockets with depths ≥5 mm while minimizing material overflow, consistent with delivery volumes reported in previous studies evaluating subgingival application of *Aloe vera* gel as a local drug delivery agent in periodontal therapy [10]. The subgingival application procedure was repeated on the 15th day to maintain therapeutic availability of the gel within the periodontal pocket. Oral hygiene instructions were reinforced for all participants throughout the study period.

The *Aloe vera* gel used in this study was prepared from fresh, mature leaves of *Aloe barbadensis Miller*, which were obtained from authenticated plant sources under institutional supervision. The outer rind of the leaves was carefully removed under aseptic conditions, and the inner mucilaginous parenchymal gel was collected and homogenized to obtain a uniform preparation. The gel was prepared immediately prior to clinical application to preserve the biological activity of its polysaccharide‐rich components and to minimize the degradation of active constituents. No additional preservatives or chemical stabilizing agents were incorporated, and the preparation represented freshly extracted whole inner leaf gel. The preparation procedure was carried out under laboratory guidance within an institutional setting, and the gel was transferred into sterile syringes for subgingival delivery. Fresh preparation before application ensured the maintenance of sterility and biological efficacy for intra‐pocket administration. The preparation protocol and intraoral use of the locally prepared *Aloe vera* gel were reviewed and approved as part of the study methodology by the Institutional Ethics Committee prior to commencement of the clinical trial. As freshly extracted inner‐leaf gel was used without dilution, the preparation corresponded to 100% *Aloe vera* mucilaginous gel in its native form.

The clinical parameters evaluated included plaque index (PI), gingival index (GI), PPD, and clinical attachment level (CAL). All measurements were recorded at baseline and at 15, 30, and 45 days using a UNC‐15 periodontal probe by a single calibrated examiner who was blinded to site allocation. Prior to study initiation, intra‐examiner calibration was performed by repeating PPD and CAL measurements at selected periodontal sites in five subjects at two separate time points 48 h apart. The intra‐class correlation coefficient (ICC) for repeated measurements was 0.88, indicating high intra‐examiner reliability.

The sample size was estimated based on the expected difference in PPD reduction between test and control sites following adjunctive local drug delivery therapy, with a significance level of 5% and statistical power of 80%. Based on these assumptions and previously reported clinical studies evaluating subgingival application of *Aloe vera* gel as an adjunct to SRP, a minimum sample size of 30 sites per group was considered adequate. A total of 37 participants fulfilling the inclusion criteria were therefore included in the study. Statistical analysis was performed using SPSS software (Version 26.0; IBM Corp., Armonk, NY, USA). Intragroup comparisons between baseline and follow‐up measurements were performed using paired *t*‐tests, while intergroup comparisons between the test and control sites were performed using independent *t*‐tests. A *p*‐value < 0.05 was considered statistically significant.

An AI‐assisted evidence mapping approach was performed as a supplementary exploratory component to contextualize the findings of the present randomized clinical trial within the broader periodontal literature. Relevant publications between 2010 and 2025 were identified through structured searches of PubMed, Scopus, and Web of Science databases using keywords related to *Aloe vera*, herbal adjuncts, and periodontal therapy. Retrieved studies were screened for relevance to the adjunctive use of *Aloe vera* in nonsurgical periodontal treatment. Computational text mining techniques were then applied to identify dominant thematic clusters, including anti‐inflammatory mechanisms, antimicrobial effects, wound‐healing activity, formulation strategies, and clinical trial outcomes. This evidence mapping procedure was performed solely for literature pattern identification and did not influence study design, data collection, statistical analysis, or interpretation of primary clinical outcomes.

## 3. Results

### 3.1. Clinical Findings

All 37 participants completed the 45‐day follow‐up period, and no adverse events were reported during the study. Baseline values of PI, GI, PPD, and CAL were comparable between the test and control sites, with no statistically significant differences in baseline measurements (Table 1).

**Table 1:** Intragroup comparison of clinical parameters at baseline and follow‐up (mean ± SD).

Parameter	Group	Baseline	15 days	30 days	45 days	*p*‐Value (intragroup)
PI	SRP + *Aloe vera*	1.89 ± 0.32	1.42 ± 0.29	1.18 ± 0.26	1.07 ± 0.23	<0.001
	SRP alone	1.85 ± 0.35	1.48 ± 0.31	1.21 ± 0.27	1.10 ± 0.25	<0.001
GI	SRP + *Aloe vera*	1.76 ± 0.28	1.32 ± 0.23	1.09 ± 0.21	1.01 ± 0.18	<0.001
	SRP alone	1.78 ± 0.27	1.38 ± 0.25	1.13 ± 0.22	1.06 ± 0.20	<0.001
PPD (mm)	SRP + *Aloe vera*	5.47 ± 0.61	4.23 ± 0.52	3.98 ± 0.48	3.84 ± 0.44	<0.001
	SRP alone	5.41 ± 0.59	4.72 ± 0.55	4.35 ± 0.49	4.29 ± 0.46	<0.001
CAL (mm)	SRP + *Aloe vera*	5.93 ± 0.64	5.12 ± 0.59	4.86 ± 0.52	4.69 ± 0.49	<0.001
	SRP alone	5.89 ± 0.62	5.47 ± 0.58	5.23 ± 0.53	5.08 ± 0.50	<0.001

*Note:* Both groups showed statistically significant intragroup improvement over time.

**Figure 1:**
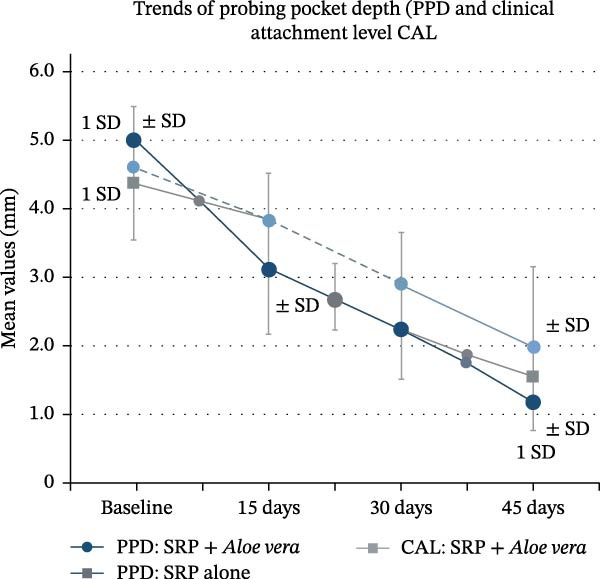
Line graph illustrating the trends in probing pocket depth (PPD) reduction and clinical attachment level (CAL) gain over the 45‐day evaluation period for both the test (SRP + *Aloe vera* gel) and control (SRP alone) groups.

Both treatment conditions demonstrated statistically significant intragroup improvements in PI, GI, PPD, and CAL over the 45‐day evaluation period (*p* < 0.001; Figure 1). At 45 days, test sites treated with SRP combined with locally delivered *Aloe vera* gel demonstrated a greater mean reduction in PPD (0.45 ± 0.09 mm) compared with control sites treated with SRP alone (*p* = 0.02). Similarly, a significantly greater mean gain in CAL was observed at the test sites (0.39 ± 0.12 mm) compared with the control sites (*p* = 0.03) (Tables 2 and 3).

**Table 2:** Intergroup comparison of clinical parameters at 45 days (mean ± SD).

Parameter	SRP + *Aloe vera*	SRP alone	Mean difference	*p*‐Value
PI	1.07 ± 0.23	1.10 ± 0.25	0.03	0.41 (NS)
GI	1.01 ± 0.18	1.06 ± 0.20	0.05	0.29 (NS)
PPD (mm)	3.84 ± 0.44	4.29 ± 0.46	0.45	0.02 ^∗^
CAL (mm)	4.69 ± 0.49	5.08 ± 0.50	0.39	0.03 ^∗^

Abbreviation: NS, not significant.

^∗^Statistically significant (*p* < 0.05).

**Table 3:** Intergroup comparison of probing pocket depth (PPD) reduction and clinical attachment level (CAL) gain at 45 days.

Clinical parameter	Group A (SRP + *Aloe vera*) mean ± SD	Group B (SRP alone) mean ± SD	*p*‐ Value
PPD reduction (mm)	0.45 ± 0.09	0.30 ± 0.08	0.02 ^∗^
CAL gain (mm)	0.39 ± 0.12	0.26 ± 0.10	0.03 ^∗^

*Note:* Group A showed significantly greater reduction in PPD and gain in CAL compared with Group B at 45 days.

^∗^Statistically significant (*p* < 0.05).

**Figure 2:**
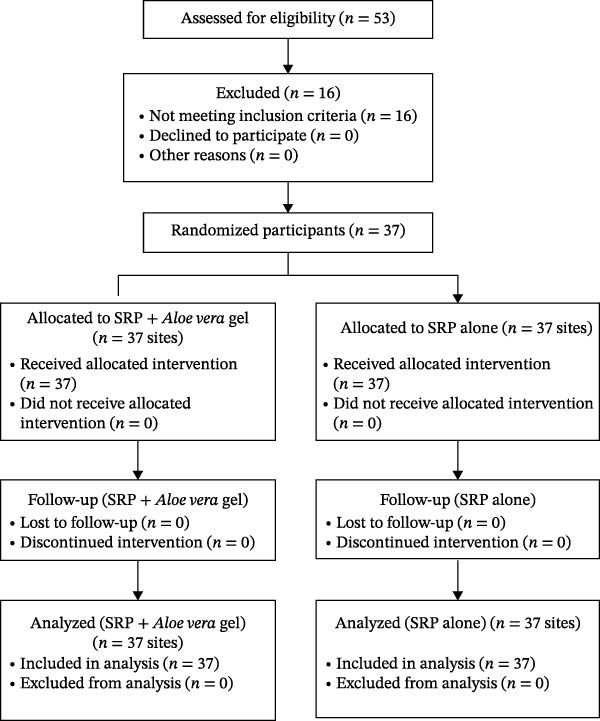
CONSORT flow diagram illustrating participant enrollment, allocation, follow‐up, and analysis for the split‐mouth randomized clinical trial evaluating adjunctive *Aloe vera* gel.

Comparisons of PI and GI between the test and control sites did not demonstrate statistically significant differences at any evaluation time point (*p* > 0.05). Trends in PPD reduction and CAL gain over time for both treatment conditions are illustrated in Figure 2.

### 3.2. AI‐Based Evidence Mapping Results

The AI‐assisted evidence mapping retrieved 57 eligible publications related to the use of *Aloe vera* in periodontal therapy published between 2010 and 2025. Topic modeling and clustering analysis identified five dominant thematic clusters: anti‐inflammatory mechanisms, antimicrobial activity against periodontal pathogens, wound healing and collagen modulation, formulation and delivery systems, and clinical trials with meta‐analyses.

**Figure 3:**
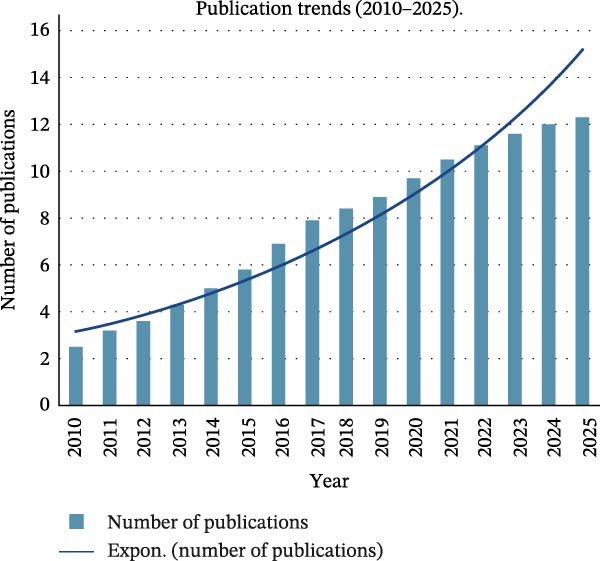
Artificial intelligence‐assisted evidence mapping results displaying publication trends related to the use of *Aloe vera* in periodontal therapy from 2010 to 2025.

Temporal trend analysis demonstrated a marked increase in publications after 2018, with recent studies focusing on translational outcomes and combination formulations (Figure 3). These thematic patterns provide contextual support for the clinical findings observed in the present trial.

## 4. Discussion

**Figure 4:**
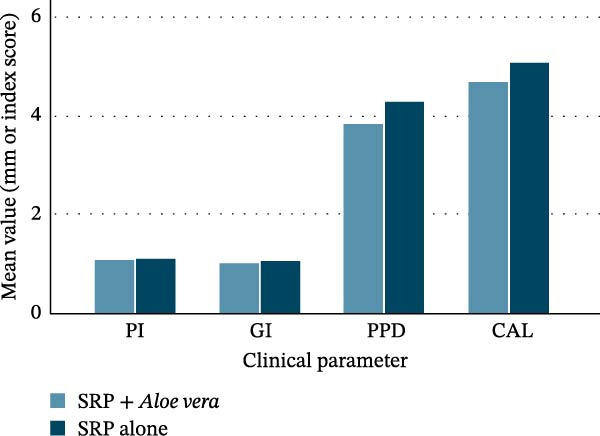
Bar chart comparing the mean values of the evaluated clinical parameters (plaque index [PI], gingival index [GI], probing pocket depth [PPD], and clinical attachment level [CAL]) between the test and control groups.

This randomized split‐mouth clinical study evaluated the adjunctive effect of locally delivered *Aloe vera* gel when used in combination with SRP in patients with stage II–III, Grade B periodontitis. The results demonstrated statistically significant reductions in plaque accumulation, gingival inflammation, PPD, and CAL in both treatment groups over the study period. However, sites receiving adjunctive subgingival *Aloe vera* gel showed greater improvement in PPD reduction and CAL gain compared with sites treated with SRP alone. In contrast to several previous studies that predominantly employed commercially available formulations or single‐application delivery protocols, the present study evaluated repeated subgingival placement of freshly prepared *Aloe vera* gel with controlled retention using periodontal dressing. This protocol was designed to enhance local bioavailability and therapeutic persistence within periodontal pockets, thereby contributing additional evidence regarding protocol‐dependent variability in adjunctive herbal periodontal therapy (Figure 4).

The intragroup improvements observed in both treatment groups are consistent with the well‐ established effectiveness of SRP in nonsurgical periodontal therapy [2, 3]. Mechanical debridement disrupts the subgingival biofilm, reduces bacterial load, and facilitates resolution of inflammation, thereby promoting periodontal healing. The absence of statistically significant intergroup differences in PI and GI may be attributed to the reinforcement of oral hygiene instructions and comparable plaque control measures followed by all participants throughout the study period.

The greater reduction in PPD and improvement in CAL observed in the *Aloe vera* group are consistent with previous clinical investigations reporting beneficial effects of *Aloe vera* as a locally delivered adjunctive therapeutic agent in periodontal treatment [10, 15, 16]. The therapeutic potential of *Aloe vera* has been attributed to its anti‐inflammatory, antimicrobial, antioxidant, and wound‐healing properties. Biologically active constituents such as acemannan, anthraquinones, and salicylic acid have been shown to modulate inflammatory pathways, enhance fibroblast proliferation, and stimulate collagen synthesis, thereby supporting periodontal tissue repair and regeneration [7, 15]. These biological mechanisms provide a plausible explanation for the additional improvements observed in periodontal pocket reduction and attachment gain in the test sites.

Similar findings have been reported by Bhat et al., who demonstrated significant reductions in PPD and gingival inflammation following subgingival application of *Aloe vera* gel as an adjunct to SRP [10]. Likewise, Timothy and Rajasekar reported significant improvement in CALs in sites treated with adjunctive *Aloe vera* compared with SRP alone [15]. The present study

corroborates these findings and further supports the potential role of repeated localized application of *Aloe vera* gel in enhancing short‐term clinical periodontal outcomes. More recent clinical evidence has also demonstrated adjunctive anti‐inflammatory benefits of locally delivered *Aloe vera* in periodontal therapy, further supporting its biological and therapeutic relevance [12, 17]. In addition, systematic evaluations have reported modest but clinically meaningful improvements in periodontal outcomes with adjunctive *Aloe vera* use while emphasizing heterogeneity related to differences in formulation characteristics, delivery strategies, and follow‐up duration [13].

Conversely, some studies evaluating herbal adjunctive therapies have reported only modest or nonsignificant benefits compared with conventional nonsurgical periodontal therapy alone [13, 18, 19]. Variability in clinical outcomes across studies may be attributed to differences in formulation characteristics, concentration of active constituents, delivery techniques, application frequency, retention strategies, and duration of follow‐up, all of which influence local bioavailability and therapeutic persistence within periodontal pockets. The repeated subgingival application protocol with controlled retention employed in the present study was specifically designed to address some of these methodological limitations and may partly explain the greater improvements observed in PPD and CAL at test sites.

The artificial intelligence‐assisted evidence mapping performed in this study provided a structured overview of thematic trends within contemporary literature on the adjunctive use of *Aloe vera* in periodontal therapy. The identification of research clusters related to anti‐inflammatory activity, antimicrobial effects, wound‐healing mechanisms, formulation strategies, and clinical outcomes supports the biological plausibility of the adjunctive effects observed in the present investigation. Importantly, this analytical component served solely as a contextual interpretive framework and did not influence study design, intervention allocation, data collection, statistical analysis, or interpretation of primary randomized clinical trial outcomes [14].

Despite the strengths of the split‐mouth randomized design, examiner calibration, participant blinding, and the standardized intervention protocol, certain limitations should be considered when interpreting the findings. The relatively short follow‐up duration limits conclusions regarding the long‐term stability of the clinical improvements observed. In addition, microbiological and biochemical parameters were not evaluated, which may have provided deeper mechanistic insight into the therapeutic effects of adjunctive *Aloe vera*. Future longitudinal randomized clinical trials incorporating microbial, immunological, and biomarker‐based assessments are therefore warranted to better characterize the therapeutic potential of locally delivered herbal adjuncts in periodontal management.

Within these limitations, the findings of the present study suggest that adjunctive subgingival application of freshly prepared *Aloe vera* gel may serve as a safe, economical, and biologically plausible supplementary approach to conventional SRP in the management of stage II–III, Grade B periodontitis.

## 5. Conclusion

In this randomized split‐mouth clinical trial, adjunctive subgingival application of freshly prepared *Aloe vera* gel in combination with SRP resulted in greater reduction in PPD and improved clinical attachment gain compared with SRP alone. Both treatment approaches demonstrated significant improvement in plaque control and gingival inflammation, reaffirming the effectiveness of conventional nonsurgical periodontal therapy. Within the limitations of the present study, adjunctive locally delivered *Aloe vera* gel may represent a safe, economical, and biologically plausible supplementary therapeutic approach in the management of stage II–III, Grade B periodontitis. Further long‐term randomized clinical trials incorporating microbiological and biomarker assessments are warranted.

## Author Contributions

**Vinita Boloor:** conceptualization, methodology, supervision, validation, writing – review & editing. **Belim Wasim Siraj:** conceptualization, investigation, data curation, formal analysis, project administration, writing – original draft, writing – review & editing. **Rajesh Kashyap:** methodology, validation, writing – review & editing. **Haziel Jenifer:** data interpretation, validation, writing – review & editing. **Shruthy Prathap:** supervision, validation, writing – review & editing. **Abdul Naeem:** investigation, data collection, writing – review & editing.

## Funding

This research received no specific grant from any funding agency in the public, commercial, or not‐for‐profit sectors.

## Disclosure

All authors have read and approved the final version of the manuscript. The corresponding author, Dr. Belim Wasim Siraj, had full access to all of the data in this study and takes complete responsibility for the integrity of the data and the accuracy of the data analysis.

## Conflicts of Interest

The authors declare no conflicts of interest.

## Data Availability

The data that support the findings of this study are available from the corresponding author upon reasonable request.
